# Multidisciplinary approaches to managing osteoarthritis in multiple joint sites: a systematic review

**DOI:** 10.1186/s12891-016-1125-5

**Published:** 2016-07-08

**Authors:** Andrew Finney, Emma Healey, Joanne L. Jordan, Sarah Ryan, Krysia S. Dziedzic

**Affiliations:** Arthritis Research UK Primary Care Centre, Research Institute for Primary Care and Health Sciences, Keele University, Staffordshire, UK; Staffordshire Rheumatology Centre, The Haywood Hospital, Burslem, Stoke-on-Trent, UK

**Keywords:** Osteoarthritis, Multidisciplinary, Multisite, Joint pain

## Abstract

**Background:**

The National Institute for Health and Care Excellence’s Osteoarthritis (OA) guidelines recommended that future research should consider the benefits of combination therapies in people with OA across multiple joint sites. However, the clinical effectiveness of such approaches to OA management is unknown. This systematic review therefore aimed to identify the clinical and cost effectiveness of multidisciplinary approaches targeting multiple joint sites for OA in primary care.

**Methods:**

A systematic review of randomised controlled trials. Computerised bibliographic databases were searched (MEDLINE, EMBASE, CINAHL, PsychINFO, BNI, HBE, HMIC, AMED, Web of Science and Cochrane). Studies were included if they met the following criteria; a randomised controlled trial (RCT), a primary care population with OA across at least two different peripheral joint sites (multiple joint sites), and interventions undertaken by at least two different health disciplines (multidisciplinary). The Cochrane ‘Risk of Bias’ tool and PEDro were used for quality assessment of eligible studies. Clinical and cost effectiveness was determined by extracting and examining self-reported outcomes for pain, function, quality of life (QoL) and health care utilisation. The date range for the search was from database inception until August 2015.

**Results:**

The search identified 1148 individual titles of which four were included in the review. A narrative review was conducted due to the heterogeneity of the included trials. Each of the four trials used either educational or exercise interventions facilitated by a range of different health disciplines. Moderate clinical benefits on pain, function and QoL were reported across the studies. The beneficial effects of exercise generally decreased over time within all studies. Two studies were able to show a reduction in healthcare utilisation due to a reduction in visits to a physiotherapist or a reduction in x-rays and orthopaedic referrals. The intervention that showed the most promise used educational interventions delivered by GPs with reinforcement by practice nurses.

**Conclusions:**

There are currently very few studies that target multidisciplinary approaches suitable for OA across multiple joint sites, in primary care. A more consistent approach to outcome measurement in future studies of this nature should be considered to allow for better comparison.

## Background

Osteoarthritis (OA) is the most prevalent form of arthritis and a chronic condition for which there are few effective treatments [1]. OA management has been the focus of international guidelines and recommendations over the last decade [2–7]. In the UK the National Institute for Health and Care Excellence (NICE) [7] has produced guidelines for OA, providing a range of effective treatment recommendations for the peripheral joint sites of the hands, hips, knees and feet.

It has been highlighted that people with OA generally have more than one painful joint site. A UK population survey identified that 68 % of people that self-report joint pain do so in multiple sites (two or more sites from the hands, hips, knees and feet), and 1.76 million people in the UK have sought treatment for osteoarthritis in two or more sites of the body [8, 9]. However, existing guidelines have been derived from trials of OA examining therapies for single joint sites, and therefore the NICE OA Guideline Development Group [7] suggested that future research should consider combination therapies for OA in multiple joint sites. The term ‘combination therapies’ suggests packages of care that cover a wide range of interventions delivered by a wide range of health disciplines; including multidisciplinary team approaches. The effectiveness of multidisciplinary packages of care that have been shown to be suitable across multiple sites of OA in primary care is currently unknown.

An initial pilot search identified no studies that had targeted multidisciplinary interventions for individuals with multiple sites of OA. Therefore the aim of this systematic review was to identify randomised controlled trials (RCTs) of adults populations with OA across multiple joint sites, receiving primary care based interventions involving multidisciplinary packages of care that utilised the NICE core treatments for OA [7] (access to information (education), exercise, weight loss), compared to single discipline interventions, no intervention or usual care.

## Methods

The methodology used for this systematic review was in line with those set out by the Centre for Reviews and Dissemination (CRD) [10] Reporting of this systematic review is guided by the Preferred Reporting Items for Systematic Reviews and Meta-Analysis (PRISMA) checklist [11]. A review protocol is available on request from the corresponding author.

### Searches

The search strategy was customised for use in databases searchable through the UK National Health Service (NHS) Evidence portal (MEDLINE 1946-, EMBASE 1974-, CINAHL 1981-, PsychINFO 1806-, British Nursing Index 1985- (BNI), Health Business Elite 1922- (HBE), Health Management Information Consortium 1983- (HMIC), Allied and Complimentary Medicine 1985- (AMED)). The Web of Science databases 1950- and the Cochrane Library 1995- were also searched. Search terms were combined for the study design (RCTs); the condition (OA in two or more sites from the hands, hips, knees or feet); profession (primary care multidisciplinary health professionals) and the setting (primary care settings). The full list of search terms is available as an appendix (see Appendix).

The date range for the search was from the inception of the databases until August 2015. Reference list checks were undertaken of all the eligible papers identified within review, current OA guidelines and systematic reviews considering non-pharmacological interventions for OA in primary care. Medical subject headings (MESH terms) were utilised and ‘exploded’. Wild-card characters ensured that all forms of searched words were included.

### Eligibility criteria

Table 1 describes the inclusion and exclusion criteria for this review.

**Table 1:** Criteria for including studies in the review

	Inclusions
Population	Adults aged 18 years and over with OA. The study population must have OA in multiple joint sites (at least two sites from the hand, hip, knee or foot).
Intervention	Multidisciplinary interventions that target the NICE^7^ core treatments in primary care (“multidisciplinary” is defined as involving at least two different health disciplines).
Comparison	Usual care, uni-disciplinary approaches, placebo interventions or no intervention.
Outcome	Primary outcomes of interest were self-reported pain, function, QoL and health care utilisation.
Design	The study population must form part of an RCT.
	Exclusions
Population	Inflammatory arthritis; surgical interventions, specified peripheral joint pain conditions, chronic widespread pain (CWP).
Intervention	Non-randomised controlled trials. Studies not published in the English language.
Comparison	Surgical interventions.
Outcome	No measure of any of the key outcomes listed in the inclusion criteria
Design	Single discipline approaches

### Selection of eligible studies

The screening of titles was undertaken by the first reviewer (AF). If there was uncertainty regarding the eligibility of a study title the abstract was obtained and added into the next phase of the review (abstract screening phase) for further clarification.

Abstracts were then screened for eligibility by the first reviewer (AF) (Table 1.). Inclusion in the review was determined through the use of an abstract eligibility screening tool designed by AF, KD and EH. The screening tool was designed to simplify the screening process and ensure all abstracts were assessed in a uniform manner to ensure all those deemed eligible met the criteria laid down in the review protocol. The screening tool allowed the reviewers to screen abstracts for four key eligibility headings, i) RCT, ii) Eligible participants, iii) Multidisciplinary interventions, iv) Key outcomes. To test the reliability of the screening tool, it was pilot tested by three reviewers for the first 20 abstracts (AF, KD & EH). This allowed testing for appropriateness and usability and checked the level of agreement amongst the reviewers. A good level of agreement (19/20) between the reviewers allowed the remaining abstracts to be screened by the first reviewer (AF). Eligible abstracts were included in the next stage of the review (the ‘full paper’ stage) if they met all four criteria on the screening tool or if further clarification on any of the criteria was needed.

For the ‘full paper’ stage a decision was made *‘a priori’* that where there was uncertainty or disagreement on the eligibility of full papers between the first and second reviewer (AF, KD); those papers would be taken forward to be reviewed by all three reviewers (AF, KD, EH). This process yielded a final set of papers for the data extraction phase.

### Data extraction

The clinical and cost effectiveness of the interventions was determined through self-reported outcomes for pain, function, quality of life (QoL) and health care utilisation. A data extraction tool was formulated by AF specifically for the review and tailored to the review question. The extraction tool was piloted by three reviewers (AF, KD & EH) and amendments were agreed to make the tool more effective at extracting only the data required. Data extraction was undertaken independently by two reviewers (AF, KD) for all papers that met the eligibility criteria for the review, with the third reviewer (EH) extracting data from half of the eligible papers, to again check the level of agreement.

### Quality assessment/Risk of bias

The Cochrane ‘Risk of Bias’ tool [12] was utilised for quality assessment in the review (see Table 2). The Cochrane risk of bias tool is the most comprehensive approach to assessing the potential for bias in RCTs included in systematic reviews [12]. The Cochrane tool has six domains for the assessment of risk of bias. The Physiotherapy Evidence Database (PEDro) [13] was also used as it focusses on trials delivered by therapists and provides a score from eleven domains to grade each paper (Table 3).

**Table 2:** Appraisal of the four reviewed studies using the Cochrane risk of bias tool

	Hopman-Rock & Westhoff (2000) [16]	van Baar et al. (2001) [19]	Rosemann et al. (2007) [18]	Hansson et al. (2010) [17]
Sequence generation	?	+	+	?
Allocation concealment	?	+	+	+
Blinding	?	?	?	?
Incomplete outcome data	?	+	?	+
Selective outcome reporting	?	?	?	+
Other sources of bias	?	?	?	?

Key (Low risk of bias = +, High risk = −, Unclear of risk = ?)

**Table 3:** Appraisal of the four studies using PEDro scores

	Hopman-Rock & Westhoff (2000) [16]	van Baar et al. (2001) [19]	Rosemann et al. (2007) [18]	Hansson et al. (2010) [17]
1. Eligibility criteria were specified	(1)	(1)	(1)	(1)
2. Subjects were randomly allocated to groups	1	1	1	1
3. Allocation was concealed	0	1	0	1
4. The groups were similar at baseline regarding the most important prognostic indicators	1	1	1	1
5. There was blinding of all subjects	0	0	0	0
6. There was blinding of all therapists who administered the therapy	0	0	0	0
7. There was blinding of all assessors who measured at least one outcome	0	0	1	0
8. Measures of at least one key outcome were obtained from more than 85 % of the subjects initially allocated to groups	1	1	0	1
9. All subjects for whom outcome measures were available received the treatment or control condition as allocated or, where this was not the case, data for at least one key outcome was analysed by ‘intention to treat’	1	1	1	1
10. The results of between-group statistical comparisons are reported for at least one key outcome	1	1	1	1
11. The study provides both point measures and measures of variability for at least one key outcome	1	1	1	1
12. Pedro Score	6	7	6	7

1 = yes; 0 = no or unsure (No.1 does not count towards the final score)

## Results

**Fig. 1:**
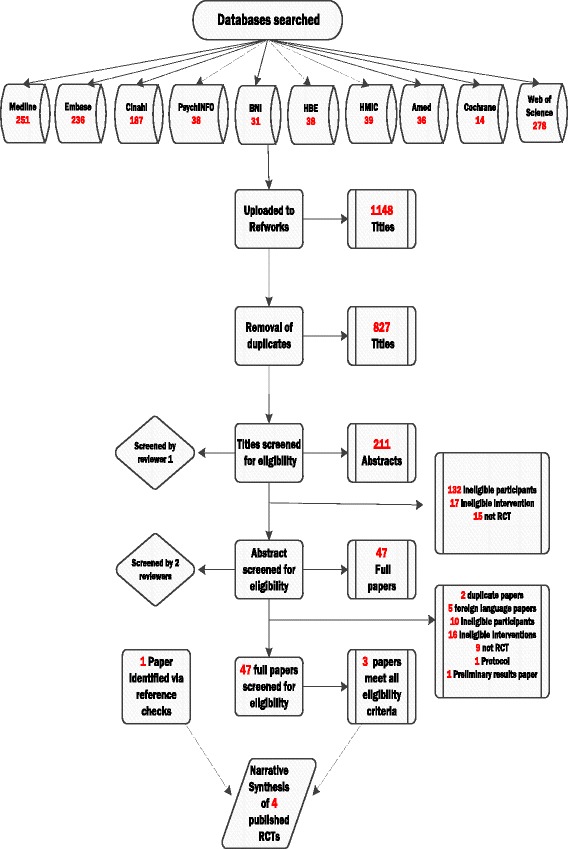
Flowchart documenting the study selection process for the review

The searches identified 1148 titles, leaving 827 after de-duplication. The screening of titles reduced the number of papers from 827 to 211. The review of abstracts resulted in the removal of 164 papers (132 involved ineligible participants, 17 did not include a suitable multidisciplinary intervention and 15 were not RCTs). Of the remaining 47 full papers, two were from the same study, five were not available in English, 10 did not include eligible participants, 16 did not use a multidisciplinary intervention and nine were not an RCT. This deductive process left five papers from three studies. Three papers were eligible for the review; one protocol paper and one preliminary results paper from two of the studies were retained for study information. Reference list checks of a relevant systematic review [14] identified one additional study that was deemed eligible. Figure 1 sets out the review process in a flowchart. An overview of the four studies included in the review and the key findings are presented in Table 4. A detailed description of the interventions tested within the four studies are presented in Table 5.

**Table 4:** An overview and key findings of the four studies included in the review

Studies & trial design	Sample size	Setting	Health disciplines	OA sites	Mean age of participants (SD)	Primary outcomes within studies	Secondary outcomes within studies	Key findings of studies
Hopman-Rock & Westhoff (2000) [16] RCT	*N* = 105 Intervention = 56 Control = 49	Single Centre (Netherlands) Primary Care	Physiotherapy, Occupational Therapy (OT) and General Practitioner	Hip or Knee	Intervention =65.4 (5.3) Control =65.2 (5.7)	IRGL self-reported pain. Pain severity (VAS)	QoL (VAS) QoL seven question sum score Activity restriction, ROM Muscle strength Observed activity restrictions Healthcare utilisation, lifestyle behaviour, BMI	Significant MANOVA effects were found for pain, QoL, quadriceps, BMI, physically active lifestyle, and visits to the physical therapist. Most effects were moderate at post-test assessment and smaller at follow-up. No effects were found for range of ROM or functional tasks
van Baar et al. (2001) [19] Single blind RCT	*N* = 201 Intervention = 99 Control = 102	Multi-Centre (Netherlands) Primary Care	General Practitioner and Physiotherapy	Hip or Knee	Intervention 68.3 (8.4) Control =67.7 (9.2)	IRGL self-reported disability, VAS pain in the past week	Observed disability, Drug use NSAIDs/paracetamol, Global perceived effect, muscle strength & ROM hip, knee, physical activity	At 24 weeks exercise treatment was associated with a small to moderate effect on pain during the past week (difference in change between the two groups −11.5 (95 % CI −19.7 to −3.3). At 36 weeks no differences were found between groups.
Rosemann et al. (2007) [18] 3-arm pragmatic cluster trial	*N* = 1021 Intervention 1 =345 Intervention 2 =344 Control = 332	Multi-Centre (Germany) Primary Care	General Practitioner and Practice nurse	Hip or Knee	Intervention 1 = 65.59 (14.68) Intervention 2 = 66.27 (15.19) Control =66.11 (15.02)	AIMS2-SF QoL, lower body, upper body, symptom & social.	IPAQ physical activity, BMI, prescriptions. Health service utilisation	Compared with the control group, for intervention group II, significant changes in the AIMS2-SF dimensions social (*p* < 0.001), symptom (*p* = 0.048), and lower body (*p* = 0.049) were identified. Radiographs (*P* = 0.031) and orthopaedic referrals (*p* = 0.044) decreased whereas prescriptions of pain relievers increased significantly.
Hansson et al. (2010) [17] Single blind RCT	*N* = 114 Intervention = 61 Control = 53	Single Centre (Sweden) Primary Care	Physiotherapy, OT, Orthopaedic Specialist, Nurse, Nutritionist	Knee, Hip or Hand	Intervention =62 (9.43) Control =63 (9.51)	EQ5D index and EQ5D VAS	ASES pain, function & other symptoms. GAT, SOLEO, SOLEC, One legged jump/raising, OA location & BMI	Significant differences between the intervention group and the control group, comparing the results at baseline and after 6 months in EuroQol-5D (*p* < 0.001) and in SOLEC (*p* = 0.02) in favour of the intervention group.

Key: *AIMS2* Arthritis Impact Measurement Scale, *ASES* Arthritis Self-Efficacy Scale, *BMI* Body Mass Index, *EQ-5D* Euro QoL, European Quality of Life measure, *GAT* Grip Ability Test, *IPAQ* International Physical Activity Questionnaire, *GP* General Practitioner, *IRGL* Impact of Rheumatic Disease on General Health and Lifestyle, *NSAIDs* Non-steroidal anti-inflammatory drugs, *QoL* Quality of Life, *ROM* Range of Movement, *VAS* Visual Analogue Scale, *SOLEO* Stand On One Leg Eyes Open, *SOLEC* Stand On One Leg Eyes Closed

**Table 5:** A detailed overview of the interventions included in the studies within the review

Studies	Intervention(s)		Control
Hopman-Rock & Westhoff (2000) [16]	Six weekly sessions lasting 2 h. First hour: Peer educator advice on Pathophysiology of OA, lifestyle and physical activity, pain management, weight reduction and diet, ergonomic and medical aspects of OA; treatments and x-rays. Questions answered by a visiting GP and OT. Second Hour: Physiotherapy lead exercise program. Education on rest and activity and the benefits of walking. Warming up exercises and relaxation exercises specific to knee and hip. Fifteen minutes of each session was spent on education about the balance between rest and activity and the types of activity. The course included the use of a pain diary and personal goal planning.		Unclear - states without intervention
van Baar et al. (2001) [19]	The patients were given exercise treatment individually by a physiotherapist in primary care (1–3 sessions per week). In addition, their GP provided patient education (including a brochure) and medication management, if necessary. One exercise protocol was used for both the hip and knee patients. It included exercises for muscle function, mobility and coordination. Instructions were also given for adaptation of the activities of daily living and home exercises.		Treatment was restricted to that given by their GP in the intervention, (patient education and medication management, if necessary).
Rosemann et al. (2007) [18]	Intervention 1: GPs received two interactive peer group meetings (8 h each) that focussed on evidence based treatment of OA in primary care (including a written summary of guidelines), arthritis self-management programs and motivational skills for working with patients. GPs were given patient education leaflets including a physical exercise programme in a booklet and on audio CD. Intervention 2: GPs received the same as Intervention 1. in addition a practice nurse was trained to monitor participants via a monthly telephone call; and to check adherence to GP prescriptions and advice and to ask about increasing pain and possible side effects of medication		Usual care
Hansson et al. (2010) [17]	The patient education programme for osteoarthritis (PEPOA). The programme lasted for 5 weeks, with group sessions once a week, 3 h for each session.		Described as ‘living as usual’
	First session	A physiotherapist and occupational therapist provided information about anatomy and physiology of pain and coping with pain. Brainstorming was used to discover what the participants found hard to do.	
	Second session	A physiotherapist provided information about exercise and physical activity and gave a practical demonstration of home-training exercises for the lower extremity. A demonstration of different kinds of orthopaedic aids for the lower extremity was also given.	
	Third session	An orthopaedic specialist, nurse and nutritionist provided information about OA and current research. Information about medications and appropriate diet were also given.	
	Fourth session	An OT provided ergonomics and practical instructions about equipment and technical aids. Feedback to the brainstorming session from session one was provided.	
	Fifth session	An OT provided information about surgery of the hand, and demonstrated the use of orthopaedic aids for hands. A practical demonstration of home training exercises for the hand was provided.	

The risk of bias was unclear in a number of sections of the eligible studies. The best way of appraising this within studies was to judge the quality and consistency of blinding, but the nature of the interventions meant that single-blinding was the only option in three studies and blinding went unreported in the cluster RCT. In using the Cochrane risk of bias tool it is hoped that ‘other sources of bias’ are identified through the use of the five main domains. It was clear that clarity of reporting the prevention of bias in clinical trials improved within the most current studies. The reporting of such studies will have been guided by the consolidated standards of reporting trials checklist (CONSORT) [15] which recommends that the key items from the Cochrane Risk of Bias tool are addressed in study publications.

A meta-analysis of the numerical data reported in the four studies was unable to be conducted as the data were considered too clinically diverse to synthesise. A narrative review of the four studies, the outcomes utilised and the results of the interventions was therefore conducted.

The overarching theme of the four papers was one of supporting self-management of OA via varying multidisciplinary approaches. The consistent interventions in each of the four studies were education and exercise. Interventions were shown to have moderate effects on QoL [16, 17], pain [16, 18, 19] and function (SOLEC test) [17]. In terms of health care utilisation, one study identified a reduction in orthopaedic referrals and x-rays [18] and one study identified a decrease in physiotherapy visits in their experimental group [16]. The clinical effects of exercise were shown to decline over time [19].

While each study used core interventions endorsed by NICE suitable for multiple sites of OA in primary care, they all had different aims and utilised different outcome measures, even when the outcomes were measuring the same attribute, i.e. pain. Each study used different disciplines to achieve a multidisciplinary approach and three of the four studies focussed solely on the hip or knee as sites of OA. Only one study targeted the hand alongside other sites [17]. No studies included the foot as a site suitable for their interventions. Each study had a higher number of female participants. The length of time participants had OA was generally unclear as it was only reported by one study [16].

### Pain

Pain was described as an outcome in three of the four studies [16, 17, 19]. The study by van Baar and colleagues [19] used a VAS of 0–100, reporting baseline pain scores and mean change at three different follow-up points (12, 24, 36 weeks). The effect size for pain at 12 weeks was 0.58 dropping to 0.36 at 24 weeks and 0.20 at the 36 week follow-up point; suggesting the benefits of their intervention on pain severity reduced over time [19].

Similarly Hopman-Rock and Westhoff [16] used a VAS of 0–100 to measure of pain intolerance and presented pre-test, post-test and follow-up results [16]. Where van Baar and colleagues identified a reduction in the improvements of pain severity over time [19], Hopman-Rock and Westhoff, demonstrated that a reduction of pain ‘post-test’ in their results had completely diminished at the 6 month follow-up point with pain severity scores worse than that at ‘pre-test’ level [16].

Rosemann and colleagues did not describe pain as a specific outcome. However, they did use the AIMS2-SF [20] within which pain is reported as ‘symptom’ [21]. Within the AIMS2-SF, there was no statistically significant improvement in pain.

Hansson and colleagues included pain dimensions from the EQ5D [22] and ASES [23] as secondary outcome measures [17]. The pain and discomfort dimension of the EQ5D significantly reduced following the intervention in those who considered their pain and discomfort to be extreme. There was no significant reduction in the pain dimension of the ASES which asked the participants whether they were able to decrease their pain [17]. None of the studies stated whether the improvements in pain were joint specific (i.e. hip or knee etc.).

### Function

Function was assessed in two studies using the IRGL [16, 19]. Reported as a measure of mobility in the Hopman-Rock and Westhoff study [16], no significant difference (*p* = 0.18) between the intervention and control group was identified. Reported as a measure of disability van Baar and colleagues, highlighted that their intervention had no effect at two follow-up time points of 12 and 24 weeks [19]. Function was also measured using a dimension of the ASES self-efficacy assessment tool in the study by Hansson and colleagues, but no significant differences between the intervention and control groups were found [17].

### Quality of life

Two studies selected QoL as a primary outcome measure, using the AIMS-2 SF [18] and the EQ5D [17]. In the cluster RCT [18] the inclusion of a practice nurse intervention (follow-up phone calls) alongside the GP intervention was responsible for an improvement in QoL due to improving ‘symptoms’ (pain). Hansson and colleagues used the EQ5D to measure five dimensions of QoL. At baseline 16 % of participants reported the pain dimension of extreme pain and discomfort; dropping to 13 % at the 6 month follow-up period. In comparison those reporting extreme pain and discomfort in the control group increased from 17 % at baseline to 21 % at the 6 month follow-up [17]. The intervention group had a higher proportion of participants improving after 6 months, yet this was not shown to be statistically significant [17].

The IRGL was also reported in one study as a secondary outcome measure for QoL, using the dimension of ‘global perceived effect’ [19]. Initial improvements in global perceived effect for the exercise group were shown to reduce over time [19].

QoL was measured using a VAS in one study and was found to be stable in a post-test assessment in the intervention group and decreased slightly in the control group [16].

### Health economics evaluation

None of the studies published a health economics evaluation of the interventions tested, however two did assess ‘Health Service Utilisation’ [16, 18]. Hopman-Rock and Westhoff measured the use of health care resources in both interventions used within their study. They found no differences in the use of medication, the number of participants consulting their GP with OA complaints, or in the mean number of GP consultations. There was a reduction in those consulting a physiotherapist at the 6 month follow-up point [16]. Rosemann and colleagues reported that there were significant reductions in orthopaedic referrals and x-rays within the intervention group that included a GP consultation and practice nurse telephone follow-up [18]. A summary of the key findings for each outcome can be seen in Table 6.

**Table 6:** Summary of key finds the main outcomes

Studies	Pain	Function	Quality of Life (QoL)	Health care utilisation
Hopman-Rock & Westhoff (2000) [16]	The IRGL pain subscale indicated that the experimental group reported fewer pain symptoms at the post-test assessment than the control group. Pain (VAS) showed a positive effect of the intervention on the experimental group	There was no improvement in IRGL mobility. No significant differences were found for extension, flexion, exorotation or endorotation of the hips and knees. The strength of knee extensors improved in both legs in post-test assessment. MANOVA showed a statistically significant improvement in strength of left knee extensor. No statistically significant improvements were seen in the functional tasks of walking, timed up-and-go, stair climbing and toe reaching as both groups improved.	Whilst QoL (VAS) remained stable at the post-test assessment in the experimental group, it had decreased in the control group. At F/U this was no longer found.	No statistically significant differences were found in the use of medication or on the number of GP consultations. Physical therapy consultations were reduced
van Baar et al. (2001) [19]	At 24 weeks (12 weeks after completion of treatment), a beneficial effect was seen for pain during the past week. Compared with the post-treatment level (week 12) the effect size had declined to 0.36, indicating a small to moderate effect.	At 24 weeks no effects were found for self-reported disability, muscle strength, and range of motion. Similar effects were found at week 36.	_	There was a reduction in the use of paracetamol at 24 weeks that remained stable at 36 weeks F/U
Rosemann et al. (2007) [18]	Statistically significant improvements were seen for the ‘Symptom’ component of the AIMS2-SF in intervention group 2.	No statistically significant improvements in IPAQ sores	Statistically significant improvements were shown for the lower body, symptom and social components of the AIMS2-SF	There was a statistically significant reduction in orthopaedic referrals in intervention group 2 and x-rays in intervention groups 1 & 2
Hansson et al. (2010) [17]	No improvements were shown for ASES pain scores. EQ-5D scores reduced in the experimental group.	There was no statistically significant improvement in ASES function, GAT or SOLEO but there was a statistically significant improvement on SOLEC	There was a statistically significant improvement in the EQ-5D VAS but not the EQ-5D index.	_

Key: *AIMS2* Arthritis Impact Measurement Scale, *ASES* Arthritis Self-Efficacy Scale, *EQ-5D* Euro QoL, European Quality of Life measure, *GAT* Grip Ability Test, *IPAQ* International Physical Activity Questionnaire, *IRGL* Impact of Rheumatic Disease on General Health and Lifestyle, *VAS* Visual Analogue Scale, *SOLEO* Stand On One Leg Eyes Open; *SOLEC* Stand On One Leg Eyes Closed

One observation by Hopman-Rock and Westhoff was the need to consider proactive follow-up; which was the aim of the study by Rosemann and colleagues [18]. The most effective intervention of the four studies appeared to be proactive nurse follow-up to GP consultations, where training was delivered to the health professionals rather than patients and significant improvements were seen in self-efficacy along with a reduction of orthopaedic referrals and x-rays and increased uptake of Paracetamol [18]. The findings of Rosemann and colleagues suggest the interaction of both the GP and practice nurse offered a continuing care approach above and beyond that of a GP consultation alone and more effective than the self-management programs utilised in the other studies.

## Discussion

The four studies identified in the systematic review were very clinically and methodologically diverse and a meta-analysis was therefore not undertaken. Multidisciplinary approaches with education and exercise were the primary interventions for self-management of OA and overall moderate improvements were shown for pain, function, QoL with reduced orthopaedic referrals and x-rays. The review aimed to be inclusive in its approach to the eligibility of studies. Two sites of OA were classed as ‘multiple sites’ and a minimum of two health disciplines was considered multidisciplinary. Additional trial protocols and trial development publications were identified in the search [24, 25] from the four studies in the review but there was limited evidence for effective multidisciplinary packages for implementing core OA recommendations in primary care.

Clinical OA guidelines have emphasised that treatments for OA are suitable for all peripheral joint sites [3–7] so if core interventions could be successfully implemented for multisite OA, this could have a significant impact on OA pain and disability [26, 27]. Outcomes often attributed to single site pain disorders are explained, at least in part, by multisite pain or pain elsewhere [28, 29] and the disappointing results for the management of OA in single sites may be explained by other painful joint sites [30]. To evaluate the true impact of an OA intervention the number of sites of OA should be either reported or adjusted for in the analysis.

Our findings are comparable to those of Cuperus et al. [31] where a multidisciplinary face-to-face treatment program was compared to a telephone based treatment program in secondary care for generalised osteoarthritis (GOA). Results were generally inconclusive, with no differences observed between groups for the primary outcome.

A weakness of this review was the lack of eligible primary care studies of interventions in multiple joint sites and the complexity of the interventions meant that the ‘exact ingredient’ of the intervention was often hard to specify with issues in developing, describing, and reproducing interventions not fully reported.

The Cochrane risk of bias check list and the PEDro scale [12, 13] identified a number of ‘unclear’ categories when rating the success of blinding rendering judgement on the quality of the studies difficult. Findings would have also been easier to synthesise with consistency in outcomes measured. Two outcomes that could have been standardised in each study were pain e.g. intensity, severity, site, frequency, or duration - yet only two of the four studies considered pain to be a primary outcome [16, 19] - and function e.g. Western Ontario and McMaster Universities Osteoarthritis Index (WOMAC) [32].

In summary the review was unable to determine the clinical and cost effectiveness of multidisciplinary interventions in primary care for OA in multiple sites. The one intervention that showed most promise was a GP consultation delivering OA education and exercise advice with telephone follow-up with practice nurses. Whether this type of intervention can be successfully implemented in primary care systems is yet to be determined.

## Conclusions

This systematic review identified four studies that used either educational or exercise interventions via various multidisciplinary team approaches to improve clinical and cost effectiveness outcomes suitable for OA across multiple joint sites. However, a meta-analysis was unachievable due to the heterogeneous nature of the studies.

A consistent approach to outcome measurement in future studies of OA across multiple sites is needed as there is limited consensus on outcome measures at present, leading to greater heterogeneity across studies. A narrative review found that only one study was able to report significant improvements in outcomes that were sustainable over time.

## Abbreviations

AIMS2, Arthritis Impact Measurement Scale; AMED, Allied and Complimentary Medicine; ASES, Arthritis Self-Efficacy Scale; BMI, body mass index; BNI, British Nursing Index; CONSORT, Consolidated Standards of Reporting Trials; CRD, Centre of Reviews and Dissemination; EQ-5D, Euro QoL five dimension; GAT, Grip Ability Test; GOA, General Osteoarthritis; GP, General Practitioner; HBE, Health Business Elite; HMIC, Health Management Information Consortium; ICOAP, The Intermittent and Constant Pain Score; IPAQ, International Physical Activity Questionnaire; IRGL, Impact of Rheumatic Disease on General Health and Lifestyle; MESH, medical subject headings; NICE, National Institute for Health and Care Excellence; NSAIDs, Non-steroidal anti-inflammatory drugs; OA, osteoarthritis; OARSI, Osteoarthritis Research Society International; OMERACT, Outcome Measures in Rheumatology; QoL, quality of life; RCT, randomised controlled trial; ROM, range of movement; SOLEC, stand on one leg eyes closed; SOLEO, stand on one leg eyes open; VAS, visual analogue scale; WOMAC, The Western Ontario and McMaster Universities Arthritis Index

## Appendix

**Table 7:** Full search terms for each database. Full search terms provided for ten searched databases

Database	Search	Results
AMED	exp RANDOMIZED CONTROLLED TRIALS/OR exp TREATMENT OUTCOME/OR exp COMPARATIVE STUDY/OR exp "DELIVERY OF HEALTH CARE"/"controlled clinical trial".ti,ab randomized.ab placebo.ab exp CLINICAL TRIALS/randomly.ab trial.ti,ab 1 OR 2 OR 3 OR 4 OR 5 OR 6 OR 7 exp ANIMALS/NOT humans.sh 8 not 9 exp OSTEOARTHRITIS/osteoarthritis.ti,ab OA.ti,ab ((degenerative adj2 arthritis)).ti,ab((joint adj3 pain)).ti,ab((knee adj3 pain)).ti,ab((hip adj3 pain)).ti,ab((hand adj3 pain)).ti,ab((foot adj3 pain)).ti,ab 11 OR 12 OR 13 OR 14 OR 15 OR 16 OR 17 OR 18 OR 19 10 AND 20 multidisciplinar*.ti,ab interdisciplinar*.ti,ab multiprofessional*.ti,abAMEDmultimodal*.ti,ab exp PATIENT CARE TEAM/"self-manage*".ti,ab exp PATIENT CARE MANAGEMENT/"patient education".ti,ab ((pain clinics OR pain service OR pain relief)).ti,ab exp OCCUPATIONAL THERAPY/"occupational therap*".ti,ab"physiotherap*".ti,ab exp PHYSIOTHERAPY/OR exp PHYSICAL THERAPY MODALITIES/exp TREATMENT OUTCOME/nurse*.ti,ab exp NURSING/pharmac*.ti,ab exp PHARMACOLOGY/OR exp COMPLEMENTARY THERAPIES/"non pharmacol*".ti,ab exp DIET THERAPY/Dietetic*.ti,ab exp OSTEOPATHY/osteopath*.ti,ab exp CHIROPRACTIC/chiroprac*.ti,ab "clinical psychol*".ti,ab exp PODIATRY/podiat*.ti,ab 22 OR 23 OR 24 OR 25 OR 26 OR 27 OR 28 OR 29 OR 30 OR 31 OR 32 OR 33 OR 34 OR 35 OR 36 OR 37 OR 38 OR 39 OR 40 OR 41 OR 42 OR 43 OR 44 OR 45 OR 46 OR 47 OR 48 OR 49 21 AND 50 exp FAMILY PRACTICE/exp PRIMARY HEALTH CARE/exp COMMUNITY HEALTH SERVICES/"general pract*".ti,ab ((primary adj2 care)).ti,ab ((secondary adj2 care)).ti,ab "family pract*".ti,ab "family physician*".ti,ab "family doctor*".ti,ab GP.ti,ab exp AMBULATORY CARE/((ambulatory adj2 care)).ti,ab "community health service*".ti,ab "community health care*".ti,ab "outpatients clinics".ti,ab 52 OR 53 OR 54 OR 55 OR 56 OR 57 OR 58 OR 59 OR 60 OR 61 OR 62 OR 63 OR 64 OR 65 OR 66 51 AND 67	36
BNI	((randomized controlled trial)).ti,ab ((randomised controlled trial)).ti,ab RCT.ti,ab randomised.ti,ab randomized.ti,ab randomly.ab placebo.ab trial.ti,ab 1 OR 2 OR 3 OR 4 OR 5 OR 6 OR 7 OR 8 exp "ARTHRITIS AND RHEUMATISM"/OR exp JOINT DISORDERS/exp "ARTHRITIS AND RHEUMATISM"/OR exp JOINT DISORDERS/osteoarthritis.ti,ab OA.ti,ab ((degenerative arthritis)).ti,ab ((joint pain)).ti,ab ((knee pain)).ti,ab ((hip pain)).ti,ab ((hand pain)).ti,ab ((foot pain)).ti,ab 10 OR 11 OR 12 OR 13 OR 14 OR 15 OR 16 OR 17 OR 18 9 AND 19 exp MULTIDISCIPLINARY TEAMS/OR exp INTERPROFESSIONAL RELATIONS/OR exp "CONTINUITY OF CARE"/interdisciplinary.ti,ab multiprofessional*.ti,ab multimodal*.ti,ab ((Patient care team)).ti,ab ((self manage*)).ti,ab ((patient care management)).ti,ab ((patient education)).ti,ab ((pain clinics)).ti,ab exp "PAIN AND PAIN MANAGEMENT"/OR exp ALTERNATIVE THERAPIES/exp OCCUPATIONAL THERAPY/((occupational therap*)).ti,ab exp PHYSIOTHERAPY/physiotherap*.ti,ab ((treatment outcome)).ti,ab exp NURSE PATIENT RELATIONS/OR exp NURSE SPECIALIST/OR exp NURSE PRACTITIONER/exp PHARMACISTS/exp PHARMACISTS/pharmac*.ti,ab ((non pharmacol*)).ti,ab OBESITY/dietician*.ti,ab dietetics.ti,ab osteopath.ti,ab osteop*.ti,ab chiropractice.ti,ab chiropractic*.ti,ab exp "FOOT CARE AND DISORDERS"/podiatry.ti,ab podiat*.ti,ab 21 OR 22 OR 23 OR 24 OR 25 OR 26 OR 27 OR 28 OR 29 OR 30 OR 31 OR 32 OR 33 OR 34 OR 35 OR 36 OR 37 OR 38 OR 39 OR 40 OR 41 OR 42 OR 43 OR 44 OR 45 OR 46 OR 47 OR 48 OR 49 20 AND 50	31
CINAHL	RANDOMIZED CONTROLLED TRIALS/OR COCHRANE LIBRARY/OR exp CLINICAL TRIALS/((controlled clinical trial)).ti,ab randomi?ed.ab placebo.ab random*.ab trial*.ti,ab 1 OR 2 OR 3 OR 4 OR 5 OR 6 ANIMALS/NOT humans.sh 7 not 8 OSTEOARTHRITIS, HIP/OR OSTEOARTHRITIS, KNEE/OR OSTEOARTHRITIS, WRIST/OR exp OSTEOARTHRITIS/osteoarthritis.ti,ab OA.ti,ab ((degenerative adj2 arthritis)).ti,ab ((joint adj3 pain)).ti,ab ((knee adj3 pain)).ti,ab ((hip adj3 pain)).ti,ab ((hand adj3 pain)).ti,ab ((foot adj3 pain)).ti,ab 10 OR 11 OR 12 OR 13 OR 14 OR 15 OR 16 OR 17 OR 18 9 AND 19 multidisciplinar*.ti,ab exp MULTIDISCIPLINARY CARE TEAM/interdisciplinar*.ti,ab multiprofessional*.ti,ab multimodal*.ti,ab exp PATIENT CARE/((patient education)).ti,ab PAIN CLINICS/OR CHRONIC PAIN/((pain sevice OR pain relief)).ti,ab OCCUPATIONAL THERAPY SERVICE/OR exp OCCUPATIONAL THERAPY/((occupational therap*)).ti,ab PHYSIOTHERAPY EVIDENCE DATABASE/physiotherap*.ti,ab exp TREATMENT OUTCOMES/nurs*.ti,ab nursing.ti,ab ((practice nurse)).ti,ab pharmac*.ti,ab PHARMACISTS/((non pharmacol*)).ti,ab DIETETICS/dietic*.ti,ab OSTEOPATHS/OR OSTEOPATHIC MEDICINE/OR MANIPULATION, OSTEOPATHIC/osteopath*.ti,ab chiroprac*.ti,ab CHIROPRACTORS/PSYCHOLOGY, CLINICAL/((clinical psycholog*)).ti,ab PODIATRY/OR PODIATRY PRACTICE/OR PODIATRIC CARE/OR PODIATRIC ASSESSMENT/OR RESEARCH, PODIATRY/podiatr*.ti,ab 21 OR 22 OR 23 OR 24 OR 25 OR 26 OR 27 OR 28 OR 29 OR 30 OR 31 OR 32 OR 33 OR 34 OR 35 OR 36 OR 37 OR 38 OR 39 OR 40 OR 41 OR 42 OR 43 OR 44 OR 45 OR 46 OR 47 OR 48 OR 49 OR 50 20 AND 51 FAMILY PRACTICE/OR MEDICAL PRACTICE/PHYSICIANS, FAMILY/PRIMARY HEALTH CARE/exp COMMUNITY HEALTH SERVICES/((general pract*)).ti,ab ((primary adj2 care)).ti,ab ((secondary adj2 care)).ti,ab ((family pract*)).ti,ab ((family physician*)).ti,ab ((family doctor*)).ti,ab GP.ti,ab AMBULATORY CARE/((ambulatory adj2 care)).ti,ab ((community health service*)).ti,ab ((community health care*)).ti,ab OUTPATIENTS/OR OUTPATIENT SERVICE/((outpatients clinics)).ti,ab 53 OR 54 OR 55 OR 56 OR 57 OR 58 OR 59 OR 60 OR 61 OR 62 OR 63 OR 64 OR 65 OR 66 OR 67 OR 68 OR 69 52 AND 70	187
EMBASE	((randomi?ed controlled trial)).ti,ab RCT.ti,ab ((clinical trial*)).ti,ab random*.ab placebo*.ab ((Multi center study)).ti,ab 1 OR 2 OR 3 OR 4 OR 5 OR 6 exp HIP OSTEOARTHRITIS/OR exp KNEE OSTEOARTHRITIS/OR exp OSTEOARTHRITIS/osteoarthritis.ti,ab OA.ti,ab ((degenerative adj2 arthritis)).ti,ab ((joint adj3 pain)).ti,ab ((knee adj3 pain)).ti,ab ((hip adj3 pain)).ti,ab ((foot adj3 pain)).ti,ab 8 OR 9 OR 10 OR 11 OR 12 OR 13 OR 14 OR 15 7 AND 16 multidisciplinar*.ti,ab interdisciplinar*.ti,ab exp INTERDISCIPLINARY RESEARCH/ multiprofessional*.ti,ab multimodal*.ti,ab exp PATIENT CARE TEAM/((self-manage*)).ti,ab ((patient care management)).ti,ab ((patient education)).ti,ab exp PAIN CLINIC/((pain service OR pain relief)).ti,ab exp OCCUPATIONAL THERAPY/((occupational therapy)).ti,ab ((occupational therapy)).ti,ab OT.ti,ab exp PHYSIOTHERAPY/Physiotherap*.ti,ab exp TREATMENT OUTCOME/PRIMARY NURSING CARE/nurs*.ti,ab ((practice nurse)).ti,ab exp "PHARMACY (SCIENCE)"/((community pharmac*)).ti,ab ((non pharmacol*)).ti,ab exp DIETETICS/dietetic*.ti,ab exp OSTEOPATHIC PHYSICIANS/OR exp OSTEOPATHY/OR exp OSTEOPATHIC PHYSICIAN/OR exp OSTEOPATH/osteopath*.ti,ab exp CHIROPODIST/OR exp CHIROPODY/OR exp CHIROPRACTIC/OR exp CHIROPRACTIC PRACTICE/OR exp CHIROPRACTOR/chiropract*.ti,ab exp CLINICAL PSYCHOLOGY/OR exp CLINICAL PSYCHOLOGIST/((clinical psycholog*)).ti,ab exp PODIATRIST/OR exp PODIATRY/podiat*.ti,ab 18 OR 19 OR 20 OR 21 OR 22 OR 23 OR 24 OR 25 OR 26 OR 27 OR 28 OR 29 OR 30 OR 31 OR 32 OR 33 OR 34 OR 35 OR 36 OR 37 OR 38 OR 39 OR 40 OR 41 OR 42 OR 43 OR 44 OR 45 OR 46 OR 47 OR 48 OR 49 OR 50 17 AND 51 exp GENERAL PRACTICE/exp PRIMARY HEALTH CARE/exp COMMUNITY BASED REHABILITATION/OR exp HEALTH CARE DELIVERY [+NT]/((community health services)).ti,ab ((primary adj2 care)).ti,ab ((secondary adj2 care)).ti,ab ((family pract*)).ti,ab ((family physician*)).ti,ab ((family doctor*)).ti,ab exp AMBULATORY CARE/((ambulatory adj2 care)).ti,ab ((community health service*)).ti,ab ((community health care*)).ti,ab exp OUTPATIENT CARE/OR exp OUTPATIENT CLINIC/OR exp OUTPATIENT CLINICS, HOSPITAL/OR exp OUTPATIENT DEPARTMENT/OR exp OUTPATIENT HEALTH CARE/OR exp OUTPATIENT SERVICE/OR exp OUTPATIENT THERAPY/OR exp OUTPATIENT UNIT/53 OR 54 OR 55 OR 56 OR 57 OR 58 OR 59 OR 60 OR 61 OR 62 OR 63 OR 64 OR 65 OR 66 52 AND 67	236
HEALTH BUSINESS ELITE	(randomi?ed controlled trial)).ti,ab ((controlled clinical trial)).ti,ab random*. placebo*.ab ((clinical trials as topic)).ti,ab trial.ti, 1 OR 2 OR 3 OR 4 OR 5 OR osteoarthritis.ti,ab OA.ti,ab ((degenerative adj2 arthritis)).ti,ab ((joint adj3 pain)).ti,ab ((knee adj3 pain)).ti,ab ((hip adj3 pain)).ti,ab ((hand adj3 pain)).ti,ab ((foot adj3 pain)).ti,ab8 OR 9 OR 10 OR 11 OR 12 OR 13 OR 14 OR 15 ELITE7 AND 16multidisciplin*.ti,abinterdisciplin*.ti,ab multiprofessional*.ti,ab multimodal.ti,ab ((patient care team)).ti, ((self manage*)).ti,ab ((patient care management)).ti,ab ((patient education)).ti,ab ((pain clinics OR pain service OR pain relief)).ti,ab ((Occupational therapy)).ti,abPhysiother*.ti,ab ((Treatment outcome)).ti, nursing.ti,ab ((practice nurse)).ti,ab Phamac*.ti,ab ((Nonpharmacol*)).ti,abDiet*.ti,ab osteopa*.ti,abchiroprac*.ti,ab ((Clinical psychol*)).ti,ab podiat*.ti,ab18 OR 19 OR 20 OR 21 OR 22 OR 23 OR 24 OR 25 OR 26 OR 27 OR 28 OR 29 OR 30 OR 31 OR 32 OR 33 OR 34 OR 35 OR 36 OR 37 OR 38 17 AND 39	38
HMIC	Exp RANDOMISED CONTROLLED TRIALS/((controlled clinical trial)).ti,ab random*.ab placebo.abHMICexp CLINICAL TRIALS/trial.ti,ab 1 OR 2 OR 3 OR 4 OR 5 OR 6HMICexp OSTEOARTHRITIS/osteoarthritis.ti,ab OA.ti,ab ((joint adj3 pain)).ti,ab ((knee adj3 pain)).ti,ab ((hip adj3 pain)).ti,ab ((hand adj3 pain)).ti,ab ((foot adj3 pain)).ti,ab 8 OR 9 OR 10 OR 11 OR 12 OR 13 OR 14 OR 15 7 AND 16	39
MEDLINE	exp RANDOMIZED CONTROLLED TRIAL/"controlled clinical trial".pt randomized.abplacebo.ab exp CLINICAL TRIALS AS TOPIC/randomly.ab trial.ti,ab 1 OR 2 OR 3 OR 4 OR 5 OR 6 OR 7exp ANIMALS/NOT humans.sh 8 not 9 exp OSTEOARTHRITIS/osteoarthritis.ti,ab OA.ti,ab ((degenerative adj2 arthritis)).ti,ab ((Joint adj3 pain)).ti,ab ((knee adj3 pain)).ti,ab ((hip adj3 pain)).ti,ab ((hand adj3 pain)).ti,ab ((foot adj3 pain)).ti,ab 11 OR 12 OR 13 OR 14 OR 15 OR 16 OR 17 OR 18 OR 19 10 AND 20 multidisciplinar*.ti,ab interdisciplinar*.ti,ab multiprofessional*.ti,ab multimodal*.ti,ab exp PATIENT CARE TEAM/"self manage*".ti,ab exp PATIENT CARE MANAGEMENT/"patient education".ti,ab exp PAIN CLINICS/((pain clinics OR pain service OR pain relief)).ti,ab exp OCCUPATIONAL THERAPY/((occupational therap*)).ti,ab physiotherap*.ti,ab exp PHYSICAL THERAPY MODALITIES/OR exp "PHYSICAL THERAPY (SPECIALTY)"/OR exp "OUTCOME ASSESSMENT (HEALTH CARE)"/exp TREATMENT OUTCOME/nurse*.ti,ab exp NURSING/pharmac*.ti,ab exp PHARMACY/"non pharmacol*".ti,abexp DIETETICS/dietetician*.ti,ab exp OSTEOPATHIC PHYSICIANS/osteopath*.ti,abexp CHIROPRACTIC/chiroprac*.ti,ab exp PSYCHOLOGY, CLINICAL/"clinical psycholog*".ti,ab exp PODIATRY/podiat*.ti,ab 22 OR 23 OR 24 OR 25 OR 26 OR 27 OR 28 OR 29 OR 30 OR 31 OR 32 OR 33 OR 34 OR 35 OR 36 OR 37 OR 38 OR 39 OR 40 OR 41 OR 42 OR 43 OR 44 OR 45 OR 46 OR 47 OR 48 OR 49 OR 50 OR 51 21 AND 52 exp FAMILY PRACTICE/exp PHYSICIANS, FAMILY/exp PRIMARY HEALTH CARE/exp COMMUNITY HEALTH SERVICES/"general pract*".ti,ab ((primary adj2 care*)).ti,ab ((secondary adj2 care*)).ti,ab"family pract*".ti,ab"family physician*".ti,ab "family doctor*".ti,ab GP.ti,ab exp AMBULATORY CARE/((ambulatory adj2 care*)).ti,ab "community health service*".ti,ab "community health care*".ti,ab exp OUTPATIENT CLINICS, HOSPITAL/OR exp OUTPATIENTS/54 OR 55 OR 56 OR 57 OR 58 OR 59 OR 60 OR 61 OR 62 OR 63 OR 64 OR 65 OR 66 OR 67 OR 6853 AND 70	251
PsycINFO	(randomi?ed controlled trial)).ti,ab ((controlled clinical trial)).ti,ab random*.ab placebo.ab exp CLINICAL TRIALS/trial.ti,ab 1 OR 2 OR 3 OR 4 OR 5 OR 6 exp CHRONIC PAIN/osteoarthritis.ti,ab OA.ti,ab ((degenerative adj2 arthritis)).ti,ab ((joint adj3 pain)).ti,ab ((knee adj3 pain)).ti,ab ((hip adj3 pain)).ti,ab ((hand adj3 pain)).ti,ab ((foot adj3 pain)).ti,ab 8 OR 9 OR 10 OR 11 OR 12 OR 13 OR 14 OR 15 OR 16 7 AND 17 multidisciplinar*.ti,ab exp INTERDISCIPLINARY TREATMENT APPROACH/OR exp TREATMENT OUTCOMES/interdisciplinar*.ti,ab multiprofessional*.ti,ab multimodal*.ti,ab ((patient care team)).ti,ab ((self manage*)).ti,ab ((patient care management)).ti,ab ((patient education)).ti,ab ((pain clinics OR pain services OR pain relief)).ti,ab exp OCCUPATIONAL THERAPY/((occupational therap*)).ti,ab (physiotherapy).ti,ab physiotherap*.ti,ab exp PHYSICAL THERAPY/exp TREATMENT OUTCOMES/exp NURSING/((practice nurs*)).ti,ab pharmac*.ti,ab exp PHARMACISTS/OR exp PHARMACOLOGY/OR exp DRUG THERAPY/non-pharmacol*.ti,ab dietician*.ti,ab dietetics.ti,ab osteopath*.ti,ab chiroprac*.ti,ab ((Clinical psychology)).ti,ab exp CLINICAL PSYCHOLOGY/podiat*.ti,ab 19 OR 20 OR 21 OR 22 OR 23 OR 24 OR 25 OR 26 OR 27 OR 28 OR 29 OR 30 OR 31 OR 32 OR 33 OR 34 OR 35 OR 36 OR 37 OR 38 OR 39 OR 40 OR 41 OR 42 OR 43 OR 44 OR 45 OR 46 18 AND 47 exp FAMILY PHYSICIANS/exp PRIMARY HEALTH CARE/((primary adj2 care)).ti,ab ((secondary adj2 care)).ti,ab ((general prac*)).ti,ab ((family pract*)).ti,ab ((family doctor*)).ti,ab GP.ti,ab exp OUTPATIENT TREATMENT/((ambulatory care)).ti,ab ((community health services)).ti,ab ((community health care)).ti,ab 49 OR 50 OR 51 OR 52 OR 53 OR 54 OR 55 OR 56 OR 57 OR 58 OR 59 OR 60 48 AND 61	38
Cochrane	MeSH descriptor Osteoarthritis explode all trees osteoarthritis (#1 OR #2) MeSH descriptor Pain Clinics explode tree 1 multidisciplinary interdisciplinary MeSH descriptor Patient Care Team explode all trees multiprofessional multi modal (#4 OR #5 OR #6 OR #7 OR #8 OR #9) (#3 AND #10)	14 Cochrane trials
Web of Science/BIOSIS	Topic = ((randomi?ed controlled trial or RCT or clinical trial)) AND Topic = ((osteoarthritis or OA or genrali?ed arthritis or degenerative arthritis or multi-site osteoarthritis)) AND Topic = ((outpatients or multidisciplinary or interdisciplinary or multiprofessional or patient care management))	278
Totals	Total of papers from all databases	1148
Totals	Total of papers remaining after de-duplication (exact match)	1030
Totals	Total of papers remaining after de-duplication (close match)	827
Totals	Total of papers remaining after ‘Title Screen’	211
Totals	Total of papers remaining after ‘Abstract Screen’	47
Totals	Total of papers remaining after ‘Full text Screen’	4
